# Characteristics and in-hospital outcomes of patients with acute coronary syndromes and heart failure in the United Arab Emirates

**DOI:** 10.1186/1756-0500-5-534

**Published:** 2012-09-26

**Authors:** Abdulla Shehab, Bayan Al-Dabbagh, Wael Almahmeed, Nazar Bustani, Nicolaas Nagelkerke, Afzal Yusufali, Adel Wassef, Mohamed Ibrahim, Azan Bin Brek

**Affiliations:** 1 Department of Internal Medicine, Faculty of Medicine and Health Sciences, United Arab Emirates University, Al Ain, United Arab Emirates; 2 Heart and Vascular Institute, Sheikh Khalifa Medical City, Abu Dhabi, United Arab Emirates; 3 Department of Community Medicine, Faculty of Medicine and Health Sciences, United Arab Emirates University, Al Ain, United Arab Emirates; 4 Dubai Heart Centre, Dubai Hospital, Dubai, United Arab Emirates; 5 Obaidallah Hospital (Saif Hospital), MOH, Ras Al-Khaimah, United Arab Emirates; 6 Department of Cardiology, Kuwaiti Hospital, Sharjah, United Arab Emirates; 7 Department of Cardiology, Rashid Hospital, Dubai, United Arab Emirates

**Keywords:** Heart failure, Acute coronary syndrome, United Arab Emirates

## Abstract

**Background:**

Heart failure (HF) is a serious complication of acute coronary syndromes (ACS), and is associated with high in-hospital mortality and poor long-term survival. The aims of this study were to describe the clinical characteristics, management and in-hospital outcomes of coronary syndrome (ACS) patients with HF in the United Arab Emirates.

**Findings:**

The study was selected from the Gulf Registry of Acute Coronary Events (Gulf RACE), a prospective multi-national, multicenter registry of patients hospitalized with ACS in six Middle East countries. The present analysis was focused on participants admitted to various hospitals in the UAE with a diagnosis of ACS in 2007 and were analyzed in terms of HF (Killip class II/III and IV) on admission. Of 1691 patients (mean age: 52.6 ± 11.7 years; 210 Females, 1481 Males) with ACS, 356 (21%) had an admission diagnosis of HF (Killip class II/III and IV). HF patients were less frequently males (19.2% vs. 34.3%; P *<* 0.001). HF was more frequently associated with hypertension (64.3% vs. 43.9%; P < 0.001), hyperlipidemia (49.4% vs. 31.8%; P < 0.001) and diabetes mellitus (DM) (51.1% vs. 36.2%; P < 0.001). HF was significantly associated with in-hospital mortality (OR = 11.821; 95% CI: 5.385-25.948; P < 0.001). In multivariate logistic regression, age, hyperlipidemia, heart rate and DM were associated with higher in-hospital HF.

**Conclusions:**

HF is observed in about 1 in 5 patients with ACS in the UAE and is associated with a significant increase in in-hospital mortality and other adverse outcomes.

## Background

Heart failure (HF) is a growing problem that puts a tremendous burden on health care facilities worldwide. Up to 70% of all patients with HF die within 5 years after their first hospital admission [1]. The most common cause of HF is coronary artery disease (CAD), including the acute coronary syndromes (ACS) of ST-segment elevation myocardial infarction (STEMI), non-STEMI (NSTEMI), and unstable angina (UA) [2]. The development of HF following ACS significantly worsens short-and long-term prognosis [3]. In developed countries, CAD is a major cause of HF, morbidity and mortality. Significant advances have been made over the last decade in therapeutic approaches to ACS, particularly the development of new antiplatelet therapies and recognition of the benefits of neurohumoral blockade. Nonetheless, ACS remains among the main causes of death in developing countries [3]. One factor contributing to the poor prognosis is development of HF and left ventricular dysfunction following myocardial infarction (MI) [4]. It is of utmost importance to identify patients at risk of developing HF at admission since it may influence immediate therapeutic choices and subsequent risk stratification strategies which consequently help improve prognosis, personalize patient care, optimize the use of healthcare resources and prevent HF and death.

The aim of this work was to describe the clinical characteristics, and in-hospital management/outcomes of ACS patients with HF in the United Arab Emirates, enrolled in the Gulf Registry of Acute Coronary Events (Gulf RACE) [5,6]. We also evaluated HF impact on in-hospital mortality.

## Methods

### Patients and data collection

Patients were selected from the Gulf Registry of Acute Coronary Events (Gulf RACE), a prospective multinational, multicentre registry of patients above 18 years of age hospitalized with the final diagnosis of ACS from various hospitals in 6 Middle Eastern countries, namely: Oman, United Arab Emirates (UAE), Qatar, Bahrain, Kuwait and Yemen [5]. Details of the Gulf RACE design and methods have been previously described [5]. There were no exclusion criteria. The recruitment in the pilot phase started from May 8, 2006, to June 6, 2006. Enrolment in the next phase of the registry started in January 29, 2007, and continued for 5 months till June 29, 2007. The present study included 1691 of 1697 patients hospitalized with an ACS across 18 hospitals in the UAE with complete data on HF (with HF: 356, 21.1%). These hospitals care for more than 85% of patients with ACS in the country. Demographic, historical, and clinical data as well as in-hospital outcomes were recorded by study physicians for all patients admitted with a diagnosis of ACS.

Definitions of the sets of variables collected from the patients, outcome parameters as well as the diagnosis of ACS types were done following the American College of Cardiology (ACC) clinical data standards [7], for example:

1. DM: history of diabetes, regardless of duration of disease, need for antidiabetic agents, or a fasting blood sugar greater than 7 mmol/l or 126 mg/dl.

2. Hypertension: documented by: 1. History of hypertension diagnosed and treated with medication, diet, and/or exercise, 2. Blood pressure greater than 140 mmHg systolic or 90 mmHg diastolic on at least 2 occasions and 3. Current use of antihypertensive pharmacological therapy.

3. Hyperlipidemia: diagnosed and/or treated by a physician. National Cholesterol Education Program criteria include documentation of the following: 1. Total cholesterol greater than 200 mg/dl (5.18 mmol/l); or 2. Low-density lipoprotein (LDL) greater than or equal to 130 mg/dl (3.37 mmol/l); or 3. High-density lipoprotein (HDL) less than 40 mg/dl (1.04 mmol/l). Treatment is also initiated if LDL is greater than 100 mg/dl (2.59 mmol/l) in patients with known coronary artery disease, and this *would* qualify as hypercholesterolemia.

4. Past revascularization: including CABG or PCI of any type (balloon angioplasty, atherectomy, stent, or other) done before the current admission with the dates noted.

5. Prior CAD: includes history of angina before the current admission and if it existed more than 2 weeks before admission and/or within 2 weeks before admission. “Angina” refers to evidence or knowledge of symptoms before this acute event described as chest pain or pressure, jaw pain, arm pain, or other equivalent discomfort suggestive of cardiac ischemia. MI: the patient at least had 1 documented previous MI before admission with the date noted.

All the patients were assigned to one of the following categories: ST-elevation myocardial infarction (STEMI), non-ST-segment elevation myocardial infarction (NSTEMI), left bundle branch block myocardial infarction (LBB MI) and UA. These definitions take into account clinical presentation, ECG findings, and the results of serum biochemical markers of myocardial necrosis (troponin and CPK-MB). Following ACS incidents after admissions, serial of cardiac enzymes were recorded and the highest (peak) values were used in our paper. Particularly, UA was defined as ACS with normal biochemical markers of necrosis. Patients were categorized at the time of hospital admission according to the classification of Killip and Kimball [8] for signs of HF. Killip class (scale I–IV) is a risk stratification tool for patients after acute myocardial infarction (AMI); a low Killip class indicates a lower likelihood of death within the first 30 days than a high Killip class. Smokers were defined as smoking cigarettes or sheesha (water pipe) within 1 month prior to index admission. A positive family history of coronary heart disease was defined as evidence of this disease in a parent, sibling, or children before the age of 55 years. The protocols of this study were approved by the Al Ain Medical District Human Research Ethics Committee, Abu Dhabi and Dubai Health Authorities and the Ministry of Health. All patients gave informed written consent to participate and care was taken to ensure data anonymity.

### Statistical analysis

All data were analyzed with SPSS statistical software version 19.0 (Chicago, Illinois, USA). The comparison of continuous variables between patients with and without HF was performed using the Student t-test. Categorical variables were compared using the Pearson’s chi-square test (or Fisher exact test for expected cells less than 5). Continuous variables with (approximately) normal distribution were expressed as mean ± SD. Multivariable backward stepwise logistic binary regression analysis was performed to estimate adjusted odds ratios (OR) of the potentially independent predictors (note: such predictors are not necessarily causes of outcome) of in-hospital HF adjusted for the following baseline covariates: age, gender, heart rate, smoking, DM, hypertension, hyperlipidemia, past revascularization including coronary artery bypasses graft surgery (CABG) or percutaneous coronary intervention (PCI) and prior coronary artery disease (CAD) including angina or myocardial infarction (MI).

Similarly, the association between HF and in-hospital mortality was examined using step-wise logistic regression models adjusting for the following covariates: age, gender, heart rate, smoking, DM, hypertension, hyperlipidemia, past revascularization including CABG or PCI and prior CAD including angina or MI. In all cases a P-value below 0.05 was considered statistically significant.

## Results

### Patient population

The records of 1691 ACS patients represent the total sample of this study. Among these, 356 patients (21.1%) had HF at hospital admission. The clinical characteristics of the study sample, by HF diagnosis at hospital admission are shown in Table 1. The mean age of the cohort was 52.5 ± 11.6 years. Patients with HF were significantly older than patients without HF (57.8 ± 13 vs. 51.2 ± 10.8 years; P < 0.001), were less likely to be male (34.3% among females vs. 19.2% among males; P < 0.001) and less likely to smoke (38.2% vs. 50.3%; P < 0.001). Risk factors for coronary artery disease were more common in patients with HF as they were more often hypertensive (64.3% vs. 43.9%), hyperlipidemic (49.4% vs. 31.8%) and diabetic (51.1% vs. 36.2%) (P < 0.001 for all comparisons). Furthermore, the HF group included a significantly higher number of patients with prior angina/MI and past PCI/CABG (50% vs. 34.2%; P < 0.001, 18.8% vs. 14.1%; P = 0.018, respectively) compared to the non-HF group. Patients with HF were slightly less likely to have STEMI on admission, but the overall distribution of admission diagnoses was not statistically different between HF and other patients. Patients with HF had a higher heart rate on presentation versus those without HF (97 ± 27.2 vs. 81.4 ± 19.8 beats/min; P < 0.001).

**Table 1:** Demographic and baseline characteristics of the studied acute coronary syndrome patients according to the presence of HF (n = 1691)

**Variable**	**HF**	**Non-HF**	**P-value**
	**(n = 356)**	**(n = 1335)**	
Age, mean ± SD, years	57.8 ± 13.0	51.2 ± 10.8	< 0.001
Male	284 (79.8)	1197 (89.7)	< 0.001
Hypertension	229 (64.3)	586 (43.9)	< 0.001
Hyperlipidemia	176 (49.4)	425 (31.8)	< 0.001
Diabetes mellitus	182 (51.1)	483 (36.2)	< 0.001
Smoking	136 (38.2)	672 (50.3)	< 0.001
Family history of CAD	39 (11.0)	221 (16.6)	0.008
Prior angina or MI	178 (50.0)	457 (34.2)	< 0.001
Past PCI or CABG	67 (18.8)	188 (14.1)	0.030
ACS diagnosis:			0.66
STEMI	141 (39.6)	572 (42.9)	
NSTEMI	81 (22.8)	302 (22.6)	
UA	131 (36.8)	453 (33.9)	
LBBB MI	3 (0.8)	8 (0.6)	
Admission characteristics:			
Killip class:			
I	-	1335 (100)	
II	221 (62.0)	-	
III	102 (28.7)	-	
IV	33 (9.3)	-	
ST deviation	287 (80.8)	998 (749)	0.021
Heart rate, mean ± SD, beats/min	97 ± 27.2	81.4 ± 19.8	< 0.001
Systolic blood pressure, mean ± SD, mm Hg	145.7 ± 40.5	141.7 ± 27.9	0.028
Diastolic blood pressure, mean ± SD, mm Hg	87.2 ± 23.7	85.6 ± 17.1	0.167
Laboratory investigations^*^:			
Peak CPK, mean ± SD, (U/l)	1269.9 ± 1930.5	1295.2 ± 1941.3	0.167
Peak CPK-MB, mean ± SD, (U/l)	99.1 ± 157.9	96.4 ± 135	0.109
Peak troponin, mean ± SD, (μg/l)^†^	19.2 ± 79.3	15.8 ± 55.9	0.581
Percent with LVEF ≤ 40%	138/282 (49)	700/959 (73)	< 0.001

^*^Values were log_10_ transformed to approximate normality for the independent sample t-test.

^†^Peak troponin I or T (depending on recruiting centre).

Figures in parentheses are percentages.

Abbreviations: *HF* heart failure, *SD* standard deviation, *CAD* coronary artery disease, *MI* myocardial infarction, *PCI* percutaneous coronary intervention, *CABG* coronary artery bypass surgery, *NSTEMI* non-ST-segment elevation myocardial infarction, *STEMI* ST-elevation myocardial infarction, *LBBB* left bundle branch block, *UA* unstable angina, *CPK* creatinine phosphokinase, *CPK-MB* creatine kinase-myocardial band isoenzyme.

#### Medical management

Table 2 outlines medication use and in-hospital management of the study patients. Compared with patients without HF, the use of aspirin, beta-blockers, calcium channel blockers, diuretics and subcutaneous insulin was significantly lower among patients with HF during their hospital treatment (Table 2). At discharge, the use of beta-blockers, calcium channel blockers, statins, nitrates, diuretics, antiarrythmic agents, digoxin and warfarin was significantly higher among patients with HF (Table 2). Moreover, Patients with HF were more likely to undergo invasive cardiac procedures than patients without HF.

**Table 2:** In-hospital and discharge management of the studied ACS patients with and without HF (n = 1691)

	**HF**	**Non-HF**	**P-value**
	**(n = 356)**	**(n = 1335)**	
In-hospital medical treatment:			
Aspirin	344 (96.6)	1328 (99.5)	< 0.001
Clopidogrel	336 (94.4)	1290 (96.6)	0.061
IV HEP	48 (13.5)	204 (15.3)	0.451
LMW HEP	311 (87.4)	1154 (86.4)	0.726
GP	132 (37.1)	534 (40.0)	0.329
BB	156 (43.8)	995 (74.5)	< 0.001
ACE	231 (64.9)	900 (67.4)	0.375
AIIRB	18 (5.1)	57 (4.3)	0.562
CCB	40 (11.2)	98 (7.3)	0.022
Statins	329 (92.4)	1268 (95)	0.068
Nitrates	268 (75.3)	934 (70.0)	0.056
Diuretics	242 (68.0)	158 (11.8)	< 0.001
SQ INS	160 (44.9)	368 (27.6)	< 0.001
IV INS	15 (4.2)	33 (2.5)	0.104
Discharge medical treatment:			
Aspirin	321 (90.2)	1293 (97.0)	< 0.001
Clopidogrel	290 (81.5)	1105 (82.8)	0.812
BB	258 (72.5)	1148 (86.0)	< 0.001
ACE	252 (70.8)	999 (74.8)	0.193
AIIRB	30 (8.4)	84 (6.3)	0.151
CCB	43 (12.1)	90 (6.7)	0.001
Statins	320 (90.9)	1273 (95.4)	0.001
Nitrates	181 (51.0)	543 (40.7)	< 0.001
Diuretics	208 (58.4)	142 (10.7)	< 0.001
Antiarrythmic agents	21 (6.0)	14 (1.0)	< 0.001
Digoxin	16 (4.5)	6 (0.4)	< 0.001
Warfarin	15 (4.2)	15 (1.1)	< 0.001
In-hospital procedures:			
Echocardiography	260 (73.0)	846 (63.4)	0.001
PCI	9 (2.5)	13 (1.0)	0.025
IABP	10 (2.8)	11 (0.8)	0.006
In-hospital outcomes:			
Death	26 (7.3)	8 (0.6)	< 0.001
Cardiogenic shock	44 (12.4)	22 (1.7)	< 0.001
Infarction	16 (4.5)	26 (2.0)	0.011
Stroke	5 (1.4)	4 (0.3)	0.023

Figures in parentheses are percentages.

Abbreviations: *DM* diabetes mellitus, *IV HEP* intravenous heparin, *LMW HEP* low molecular weight heparin, *GP* glycoprotein IIb/IIIa inhibitors, *BB* beta-blockers, *ACE* angiotensin-converting enzyme inhibitors, *AIIRB* angiotensin II receptor blockers, *CCB* calcium channel blockers, *SQ Insulin* subcutaneous insulin, *IV Insulin* intravenous insulin, *PCI* percutaneous coronary intervention, *IABP* Intra-aortic balloon pump.

### Predictors of HF

Table 3 shows that the odds of HF increases by 3.4% per year of age (OR = 1.034; 95% CI: 1.021-1.047; P < 0.001). Furthermore, patients with a history of hyperlipidemia were 1.5 times (OR = 1.539; 95% CI: 1.096-2.162; P = 0.013) more likely to present with HF than those without history of dyslipidemia. The risk of HF increased by 2.7% (OR = 1.027; 95% CI: 1.021-1.034; P < 0.001), for every 1-beat-per-minute increase in heart rate. Finally, the model also showed that having diabetes mellitus (DM) increased the risk of HF by 42% (OR = 1.425; 95% CI: 1.047-1.938; P = 0.024).

**Table 3:** Independent predictors of in-hospital HF using multivariable logistic regression

**Criteria**	**OR**	**In-hospital HF**	** P**
		** 95% CI**	
Age	1.034	1.021-1.047	< 0.001
hyperlipidemia	1.539	1.096-2.162	0.013
Heart rate	1.027	1.021-1.034	< 0.001
Prior CAD	1.322	0.949-1.841	0.100
DM	1.425	1.047-1.938	0.024

Abbreviation: *HF* heart failure, *OR* odds ratio, *CI* confidence interval, *CAD* coronary artery disease including angina or myocardial infarction *(MI)*, *DM* diabetes mellitus.

The variables that were dropped out of the multivariable logistic regression using the stepwise-backward elimination method included gender, smoking, hypertension and past revascularization (CABG/PCI).

### Heart failure and adverse outcomes including in-hospital mortality

Major bleed (n = 15) and stroke (n = 9) were rare outcomes. Stroke was significantly associated with in-hospital mortality, even after adjustment for HF (P < 0.001). Table 4 indicates that HF was significantly associated with in-hospital mortality (OR = 11.821; 95% CI: 5.385-25.948; P < 0.001). The in-hospital mortality rate for male patients was substantially lower than the mortality rate for female patients (OR = 0.419; 95% CI: 0.195-0.902; P < 0.033; using stepwise (backward selection) logistic regression).

**Table 4:** Relationship between HF and in-hospital mortality using multivariable logistic regression

**Criteria**	**OR**	**In-hospital mortality**	**P**
		** 95% CI**	
HF	11.821	5.385-25.948	< 0.001
Gender	0.419	0.195-0.902	0.033
Heart rate	1.013	1.000-1.026	0.068

Abbreviation: *HF* heart failure, *OR* odds ratio, *CI* confidence interval.

The variables that were dropped out of the multivariable logistic regression using the stepwise-backward elimination method included age, smoking, diabetes mellitus, hypertension, hyperlipidemia, past revascularization (CABG/PCI) and prior coronary artery disease (CAD) include angina or (MI).

## Discussion

The overall incidence of HF complicating ACS in our study was 21% (less so in males which represented 87.6% of the study population). This rate of HF is lower than reported in some previous studies of HF after AMI [9,10] but similar to several other ones [11-13]. Since there is no definitive diagnostic test for HF, these differences may well be due to varying definitions of HF among studies, exclusion criteria or as in the present study, inclusion of all patients with ACS, or could be due to different delays in seeking care thereby not receiving timely diagnosis or optimal therapies. It was observed that one in four ACS patients from six Middle Eastern countries in the entire GULF RACE cohort had HF [11]. In this study and using only the UAE data from the GULF RACE, we observed that one in five patients with ACS had HF during admission. This high rate of HF patients with ACS could be due to the high prevalence of DM in the region [14,15]. There is an increasing recognition that diabetic patients suffer from diabetic cardiomyopathy, including AMI and HF, which was originally described in 1972 on the basis of observations in four diabetic patients who presented with HF without evidence of hypertension, CAD, valvular or congenital heart disease [16]. Not surprisingly DM was also associated with HF in our study although our methodology precludes definitive causal interpretations. Most likely, because of the proinflammatory, and prothrombotic states associated with DM, diabetic patients with ACS are at high risk of subsequent cardiovascular events with poorer outcome and higher mortality rates [17-19]. This study also shows that HF is a common complication associated with all forms of ACS. Patients with ACS complicated by HF were more likely to die in the hospital or experience other in-hospital complications such as cardiogenic shock, infarction or stroke, although it is unclear from our logistic regression analyses whether this is because HF was the direct cause of in-hospital death (and other complications) in our patients or just a sign of substantial cardiac damage. Of note is that in-hospital mortality in our study of HF patients was 7.3%, which is relatively high. In-hospital mortality in large US registries was about 4% [20,21], whereas in the European registries it ranged from 3.8% (ESC-HF Pilot) to 6.7% (EHFS II) and 7.1% (FINN-AKVA) [22-24]. There was a lower frequency of PCI and use of beta-blockers in the patients with HF. While the lower use of beta-blockers may be explained by the difficulty of starting such therapy early in patients with HF, the reason for the low rate of cardiac catheterization in these patients is uncertain.

The strengths of this investigation include its national perspective, the complete spectrum of ACSs experienced by the large number of patients studied and the use of standardized criteria for defining ACS and hospital outcomes. However, as with any observational registry-type study, the present findings have limitations. Although this registry includes 18 hospitals throughout the UAE, participating sites may not be representative of the whole country. Furthermore, post admission variables analyses related to physician discretion (medications, interventions) may be strongly influenced by unmeasured confounders. Our analysis is also limited by the lack of adequate follow up data on long term mortality and morbidity and it is possible that some patients were misclassified according to the Killip and Kimball [8] classification for signs of HF. Finally, the standard practice management of ACS in the participating hospitals were initially, predominantly conservative. There were few PCI facility centers and hardly any primary PCI center with an established interventional cardiology program (24 h/7 days) which has an important impact upon the incidence and severity of HF.

## Conclusions

In conclusion, in this cohort of patients with ACS in the UAE, HF complicates one-fifth of cases and was associated with age, heart rate, hyperlipidemia and DM. In-hospital mortality rate was associated with HF and gender. Further studies are needed to better risk-stratify patients with HF for more analysis as well as having information about prior history of HF and outcomes of post-hospital discharge in order to optimize the use of medications and cardiovascular interventions for this serious condition.

## Competing interests

The authors declare that they have no competing interests.

## Authors’ contributions

AS and BA performed data analysis and interpretation, critically reviewed the data analysis and wrote the paper. BA, NN, and AS performed the statistical analysis. WA and NB were involved in the inception and implementation and data collection of Gulf RACE in general and the UAE data. NN, AY, AW, MI and AB were involved in the critical revision of the manuscript. All authors have read and approved the final version of the manuscript.
